# Gut Microbiota, Diet, and Chronic Diseases: The Role Played by Oxidative Stress

**DOI:** 10.1155/2019/7092032

**Published:** 2019-12-09

**Authors:** Elisardo C. Vasquez, Thiago M. C. Pereira, Manuel Campos-Toimil, Marcelo P. Baldo, Veronica A. Peotta

**Affiliations:** ^1^Pharmaceutical Sciences Graduate Program, Vila Velha University (UVV), Vila Velha, ES, Brazil; ^2^Federal Institute of Education, Science and Technology (IFES), Vila Velha, ES, Brazil; ^3^Farmacología de las Enfermedades Crónicas (CDPHARMA), Centro de Investigación en Medicina Molecular y Enfermedades Crónicas (CIMUS), Universidade de Santiago de Compostela, Santiago de Compostela, Spain; ^4^Department of Pathophysiology, State University of Montes Claros, Montes Claros, MG, Brazil; ^5^Department of Medicine, Faculdades Integradas Pitagoras, Montes Claros, MG, Brazil; ^6^Steady Family Department of Pediatrics, The University of Iowa, Iowa City, IA, USA

## 1. Introduction

While infectious diseases constituted the most serious global health issue until the middle of the 20th century [1]; nowadays, the cardiovascular and metabolic diseases are the largest contributors to global morbimortality [2]. Interestingly, if in the past centuries, bacteria were considered a potential threat to health, currently, it is well known that in normal conditions in some body compartments, such as the gut, there is a pool of microbe that is majorly composed of nonpathogenic microorganisms (“nice guys”) that are relevant to oppose the progression of several chronic diseases. Moreover, recent studies have revealed that diseases related with dysbiosis of the gut microbiota are linked with an exacerbated oxidative stress [3]. Recent data have revealed that the healthy bacteria of gut microbiota may promote a physiological cross-talk with other systems such as the brain, cardiovascular organs, and metabolic-related tissues, helping to avoid and fight hypertension and metabolic syndrome progression [3]. In parallel, other studies demonstrated that the disturbance of gut microbiota (triggered and caused by urban diets and sedentary lifestyle) may result in excessive bioavailability of reactive oxygen species (ROS) and contribute to the increase of oxidative stress [4].

**Figure 1:**
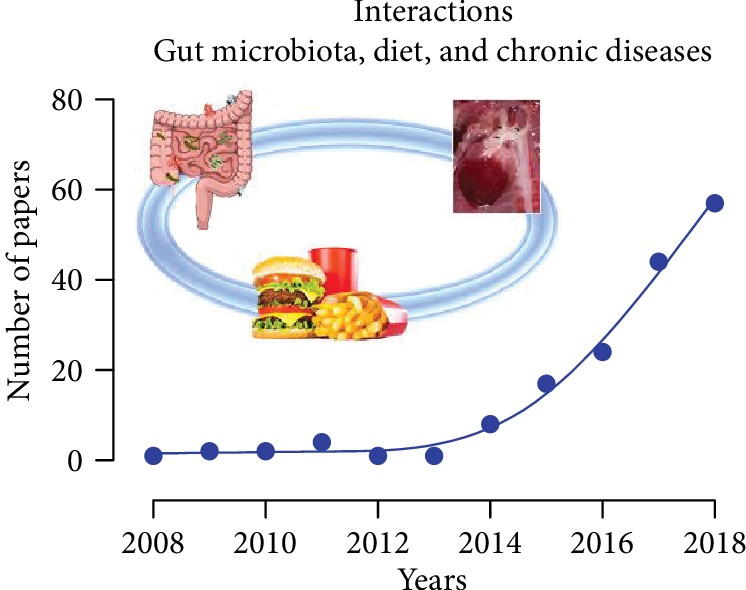
Follow-up of a decade of published papers related with the association between the three keywords: gut microbiota, diet, and chronic diseases, based on PubMed indexation. Data source: https://www.ncbi.nlm.nih.gov/pubmed/.

Therefore, the three terms used in the title of this special issue: gut microbiota, diet, and chronic diseases, that have sounded so discrepant until the last century begin to have a stronger meaning in the present century, as illustrated in Figure 1. However, a further and wide investigation is still necessary to reach a better understanding about the interactions either through the influence of microbiota and diet or through pharmacological strategies.

The special issue comprises one review article and innovative research papers that provide new insights about the potential benefits and molecular mechanisms of nutraceuticals and their antioxidant actions, as summarized below.

The review provides a state-of-the-art role showing strong evidence of the effects of the probiotic kefir in the prevention and treatment of gut dysbiosis in cardiometabolic disturbances. The authors discuss about the improvement of autonomic control of cardiovascular function and provide beneficial effects of this kind of nutraceutical in patients suffering heart failure. They also provide details and new insights about the molecular mechanisms by which kefir decreases oxidative stress in such conditions. The review contains 5 schematic figures that can be used by the readers as an updated source of knowledge about the mechanisms related with the treatment of chronic diseases by using probiotics [5].

The translational original paper presented by L. A. Chisté et al. [6] is related with novel insights about the prooxidative and genotoxic effect of sugar-sweetened soft drinks (but not zero soft drinks) and other questions that remain to be addressed concerning the consumption of commercial beverages. This innovative investigation conducted in the laboratory of Pereira and Campagnaro, using a mouse model of dyslipidemia, may effectively provide a basis for further experimental and clinical studies to explore the association between the consumption of soft drinks and metabolic diseases.

Interestingly, Chinese researchers [7] report in this issue data showing that neonatal supplementation of oxygen therapy may compromise the nephrogenesis process. The authors highlight the importance of MAPK/ERK signaling, HIF-1*α*, or catalase to protect against hyperoxia-induced oxidative damage in neonatal proximal tubules. Their study may open some news avenues that can provide the understanding of etiology cases of nephrogenesis impairment.

It is known that overproduction/accumulation of butyrate (a short-chain fatty acid of ruminal fermentation) in the bowel may promote toxic effects in the intestinal mucosa. A second group of Chinese researchers [8] present an interesting data showing that vitamin B3 (niacin) can inhibit butyrate-induced apoptosis of rumen epithelial cells. This protective effect reported by the authors is probably associated with reduced oxidative stress, inhibition of caspase-3 and p53 activation, and DNA-damage repair. The importance of that study is that it expands strategies to mitigate the damage produced by the excessive exposition of short-chain fatty acids. In the same field of research, a second group from China [9] describes the protective effects exhibited by methane-rich saline on acetic acid-induced ulcerative colitis and describes that this approach culminates with antioxidative and anti-inflammatory effects. The authors emphasize that this approach could be anti-inflammatory and antiapoptotic therapeutic alternatives through the inhibition of TLR4/NF-*κ*B/MAPK signaling pathway and promoting IL-10/JAK1/STAT3.

Considering that *Salmonella* infection is frequently observed worldwide and often is resistant to prolonged antibiotic treatment, the search for innovative antibiotics against these gram-negative bacteria deserves urgent attention. In this regard, the paper published by O. A. S. Mayorga et al. [10] demonstrates that exposition to species rich in flavonoids, the *Kalanchoe brasiliensis*, has beneficial antibacterial effects against *Salmonella* strains, by an interesting mechanism that comprises cell membrane integrity and/or permeability alterations. This discovery published in this special issue by Brazilian researchers deserves special merit because it can provide good candidates to reach the goal of new antibiotics for therapeutic applications against *Salmonella* spp.

**Figure 2:**
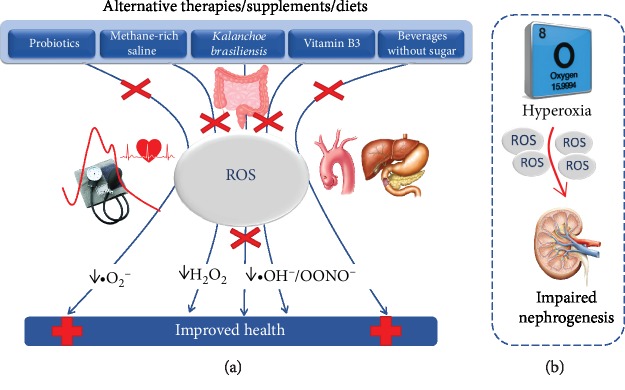
(a) Schematic diagram showing alternative therapies proposed in this special issue that exhibit antioxidative properties and become potential promissing approaches. (b) illustrates the deleterious actions of hyperoxia exposition that contributes to the worsening of the renal function and that is not yet tested with those therapies.

In conclusion, the readers will find interesting papers that were accepted and published in this special issue because even if the data are from independent researchers and laboratories, they demonstrate that different alternative therapies/supplements/diets have a molecular mechanism of action converging to anti-ROS action with distinct consequences, as illustrated in a schematic diagram (Figure 2).
